# Childhood hospitalisation with infections and later development of ankylosing spondylitis: a national case-control study

**DOI:** 10.1186/s13075-016-1141-8

**Published:** 2016-10-22

**Authors:** Ulf Lindström, Sofia Exarchou, Elisabeth Lie, Mats Dehlin, Helena Forsblad-d’Elia, Johan Askling, Lennart Jacobsson

**Affiliations:** 1 Department of Rheumatology and Inflammation Research, Institute of Medicine, Sahlgrenska Academy, University of Gothenburg, Guldhedsgatan 10A, 405 30 Gothenburg, Sweden; 2 Section of Rheumatology, Department of Clinical Sciences, Lund University, 221 85 Lund, Sweden; 3 Diakonhjemmet Hospital, P.O. Box 23 Vinderen, 0319 Oslo, Norway; 4 Institution of Public Health and Clinical Medicine/Rheumatology, Umeå University, 901 87 Umeå, Sweden; 5 Rheumatology Unit & Clinical Epidemiology Unit, Department of Medicine Solna, Karolinska Institutet, Solna, 171 76 Stockholm Sweden

**Keywords:** Ankylosing spondylitis, Epidemiology, Infections, Spondyloarthritis

## Abstract

**Background:**

The role of environmental exposures in the pathogenesis of ankylosing spondylitis (AS) remains unclear. In particular, two types of exposures have been suspected to play a role: mechanical stress and infections. The objective of this case-control study was to determine if childhood infections are associated with later development of AS.

**Methods:**

The cases with AS were identified through the Swedish national outpatient specialised-care register, based on having been given at least one AS diagnosis in the register between 2001 and 2010. Five controls per case were identified in the Swedish population register, matched at the time-point of the index case’s first spondyloarthritis diagnosis on sex, birth year, and county. All cases/controls matched prior to the age of 17 years were excluded, as well as all cases/controls given a diagnosis of reactive arthritis or juvenile arthritis at any time point, or any other diagnosis of a rheumatic disease, psoriasis, iridocyclitis, or inflammatory bowel disease before the time-point of matching. All events of hospitalisation with an infection before the age of 17 years were retrieved from the register, and categorised according to the focus of the infection. Odds ratios (ORs) and confidence intervals (CIs) were determined through conditional logistic regression analyses.

**Results:**

Of the 2453 cases with AS and 10,257 controls, 17.4 % of the cases and 16.3 % of the controls had been hospitalised with an infection before the age of 17 years (OR 1.08, 95 % CI 0.96–1.22).

Appendicitis (1.5 % cases; 2.5 % controls; OR 0.59, 95 % CI 0.41–0.83), respiratory tract infections (cases 11.2 %; controls 9.2 %; OR 1.24, 95 % CI 1.07–1.44) and, in particular, tonsillitis (cases 3.7 %; controls 2.8 %; OR 1.31, 95 % CI 1.03–1.67) were associated with AS. There were no associations between AS and any other type of infection, and the point estimates were similar in several sensitivity analyses.

**Conclusions:**

Childhood appendicitis was associated with a decreased risk, whereas respiratory tract infections were associated with an increased risk for later development of AS. These findings support a possible relationship between childhood infections and later development of AS, although the study is limited to infections resulting in inpatient care.

**Electronic supplementary material:**

The online version of this article (doi:10.1186/s13075-016-1141-8) contains supplementary material, which is available to authorized users.

## Background

An understanding of the genetic basis for ankylosing spondylitis (AS) is expanding rapidly. According to studies in twins, genetic factors, including human leukocyte antigen 27 (HLA-B27), are now thought to account for >90 % of the risk for AS [1]. In contrast, the role of environmental exposures in the aetiology of AS remains unclear. Whereas studies on twins and extended families do suggest that non-hereditary factors are likely to be involved in the pathogenesis, studies indicating a temporal relationship between environmental exposures and the development of AS are rare [2–4]. The two most prevailing theories suggest that onset of AS in genetically susceptible individuals may be triggered by either infections or by mechanical stress [5, 6], and sparse data also suggests an association with smoking [7].

Reactive arthritis, another spondyloarthritis (SpA) disease, is usually triggered by enteric or urogenital infections, but other infections, such as respiratory tract infections, can also trigger the disease onset [8–10]. Like AS, reactive arthritis is also strongly associated with HLA-B27 [11], suggesting a significant genetic overlap with AS. Similarly, in inflammatory bowel disease (IBD), enteric infections or alterations in gut flora have been implicated in the disease pathogenesis [12]. For example, in ulcerative colitis, a decreased risk for developing the disease, associated with prior appendicitis, has been described in several patient cohorts. IBD also shares a genetic overlap with AS, and while only a minority of patients with AS have a clinically overt IBD, the majority have been found to have a subclinical gut inflammation [13, 14]. The definitive role of this gut inflammation in SpA has not been determined, but it appears to share inflammatory pathways with the bone/joint inflammation [15]. Studies have also indicated differences in the gut flora between patients with AS and healthy controls, suggesting that gut dysbiosis may be involved in the disease pathogenesis [16], a finding further supported by SpA models in HLA-B27 transgenic rats, where animals reared in a germ-free environment fail to develop gut and joint inflammation [17]. In psoriasis, also related to the SpA disease group, tonsillitis, specifically with streptococci, is well known to trigger the guttate form, but is also suspected to play a role in exacerbations of plaque psoriasis [18].

In contrast to the growing data supporting a role for infections in the pathogenesis of other SpA and SpA-related diseases, no such relationship has been shown for AS, and attempts to link specific pathogens to the disease have either not been successful or not possible to replicate [16, 19, 20]. In this study, we compared the frequency of childhood infections at hospitalisation between cases later diagnosed with AS and matched population controls. Our hypothesis was that AS would be associated with childhood infections, and considering the similarities with reactive arthritis and inflammatory bowel disease, in particular with enteric and urogenital infections.

## Methods

### Setting

This is a case-control study based on four national registers in Sweden.

### Data sources

The Patient Register was initiated in 1964 as a hospitalisation register, and was expanded in 2001 with an outpatient register for specialised care [21]. The registers are considered to have an overall high validity [21].

The Population Register contains demographic data for all residents in Sweden [22].

The Prescribed Drug Register was started in July 2005, and contains anatomical therapeutic chemical (ATC) codes and administrative data on all prescribed drugs dispensed in Sweden [23].

The Swedish Rheumatology Quality Register (SRQ) contains information on anti-rheumatic treatment in Sweden. The register coverage for patients treated with tumour necrosis factor alpha inhibitors (TNFi) is 85–90 % [24].

### Study population

The AS cases were defined as having been given ≥1 diagnosis (according to the International Classification of Diseases (ICD)) of AS in the specialised care outpatient register between 2001 and 2010, being born in Sweden in 1964 or later, and having lived in Sweden at least up to the age of 17 years. The high validity of the AS diagnoses in the register has been described previously [25].

Five controls per case were retrieved from the population register, matched on sex, birth year, and county of birth, at the time of the index cases’ first registered diagnosis of SpA, and with the same requirement of having lived in Sweden at least until the age of 17 years.

In order to minimise the risk for the cases and controls already having an inflammatory disorder before the age of 17 years, all cases receiving their first registered diagnosis of SpA before the age of 17 years, as well as all cases and controls given a diagnosis of juvenile arthritis or reactive arthritis at any time, or a diagnosis of any other rheumatic disease, IBD, psoriasis, or iridocyclitis before the time-point of the match were excluded (for ICD and ATC codes see Additional file 1).

### Exposure data

For cases and controls, all ICD codes corresponding to an infection given at the time of discharge from inpatient care before the age of 17 years were identified. The data were categorised according to the primary focus of the infection and, as a supplement to enteric infections and respiratory tract infections, data for appendectomy and tonsillectomy were also collected. Data were also retrieved from the prescribed drugs register and the SRQ regarding use of immunosuppressive drugs and antibiotics in 2006–2011.

### Statistics

Odds ratios (ORs) with 95 % confidence intervals (CIs) were computed for the association between the different infectious exposures and AS using simple conditional logistic regression.

SAS 9.4 was used for aggregation of data and SPSS 21 for statistical analysis.

### Sensitivity analyses and stratification

Three a priori defined sensitivity analyses were performed. The first included only cases matched at an age of >26 years. The purpose of this analysis was to further separate the time period of exposure to the infections from the time-point of receiving the first SpA diagnosis, giving a minimum of a 10-year lag period between the exposures and the outcome, as recorded in the register.

The second included only cases treated with methotrexate, sulphasalazine, and/or TNFi, with the intention of probably selecting a more severe AS phenotype.

The third included only cases/controls matched in 2007–2011, excluding all cases and controls treated with immunosuppressive or cytostatic drugs, as well as specific drugs for IBD, during the year prior to matching. The purpose of this was to further eliminate possible cases and controls with an inflammatory disorder before the match. The limitation to cases matched after 2007 was due to the fact that the Prescribed Drug Register was started in July 2005. Comparisons of the frequencies of dispensed prescriptions of antibiotics in the year prior to match between cases and controls were also performed using Fisher’s exact test.

An additional, fourth, post-hoc sensitivity analysis was also performed, excluding cases and controls with a diagnosis of IBD between the time-point of match and up until 2 years after the first AS diagnosis, the rationale for this being the previously described association between appendicitis and a lower risk for ulcerative colitis [26, 27].

Two stratified analyses were also performed; the first stratified on median age at first SpA diagnosis, and the second for median age at appendectomy and tonsillectomy. For the latter analysis, the procedures of appendectomy and tonsillectomy were chosen, rather than the codes for the corresponding infections/inflammations, in order to avoid cases and controls with multiple registered diagnoses at different ages. For the few cases and controls with more than one registered tonsillectomy procedure, the first was used for the analysis.

## Results

### Cases and controls

We identified 2453 cases with AS (64 % men) and 10,257 controls (mean 4.3 controls per case). Demographics, AS-related disease manifestations, and pharmacological treatment are shown in Table 1.

**Table 1:** Demographics, ankylosing spondylitis (AS)-related inflammatory diseases, and pharmacological treatment in cases and controls

Demographics	Cases (*n* = 2453)	Controls (*n* = 10,257)
Men, *n* (%)	1569 (64)	6587 (64)
Year of birth, median (min–max)	1971 (1964–1992)	1971 (1964–1992)
Age at first SpA-diagnosis (years), mean, SD (min–max)	30, 6.2 (17–46)	30, 6.2 (17–46)
AS-related inflammatory diseases^a^		
Iridocyclitis, *n* (%)	453 (19)	45 (0)
Inflammatory bowel disease, n (%)	102 (4)	37 (0)
Psoriasis, *n* (%)	122 (5)	79 (1)
Pharmacological treatment in 2011^b^		
NSAID, *n* (%)	1279 (52)	9 (0)
TNF inhibitor, *n* (%)	553 (23)	NA^c^
Methotrexate, *n* (%)	169 (7)	0
Sulphasalazine, *n* (%)	263 (11)	1 (0)

^a^AS-related inflammatory diseases are given as cumulative incidence until 2 years after first AS diagnosis for the cases and their matched controls

^b^Based on prescriptions in the prescribed drugs register during 2011, apart from the tumour necrosis factor alpha (TNF) inhibitor infliximab for which data were based on recorded treatment in the Swedish Rheumatology Quality Register (SRQ) during 2011

^c^Total TNF inhibitor exposure was not possible to determine for controls since treatment with infliximab for nonrheumatic diseases cannot be detected through either the prescribed drugs register or SRQ. The frequency of any subcutaneous TNF inhibitors for controls during 2011 was *n* = 2

*NA* Not available, *NSAID* nonsteroidal anti-inflammatory drug, *SpA* spondyloarthritis

### Analyses

**Fig. 1:**
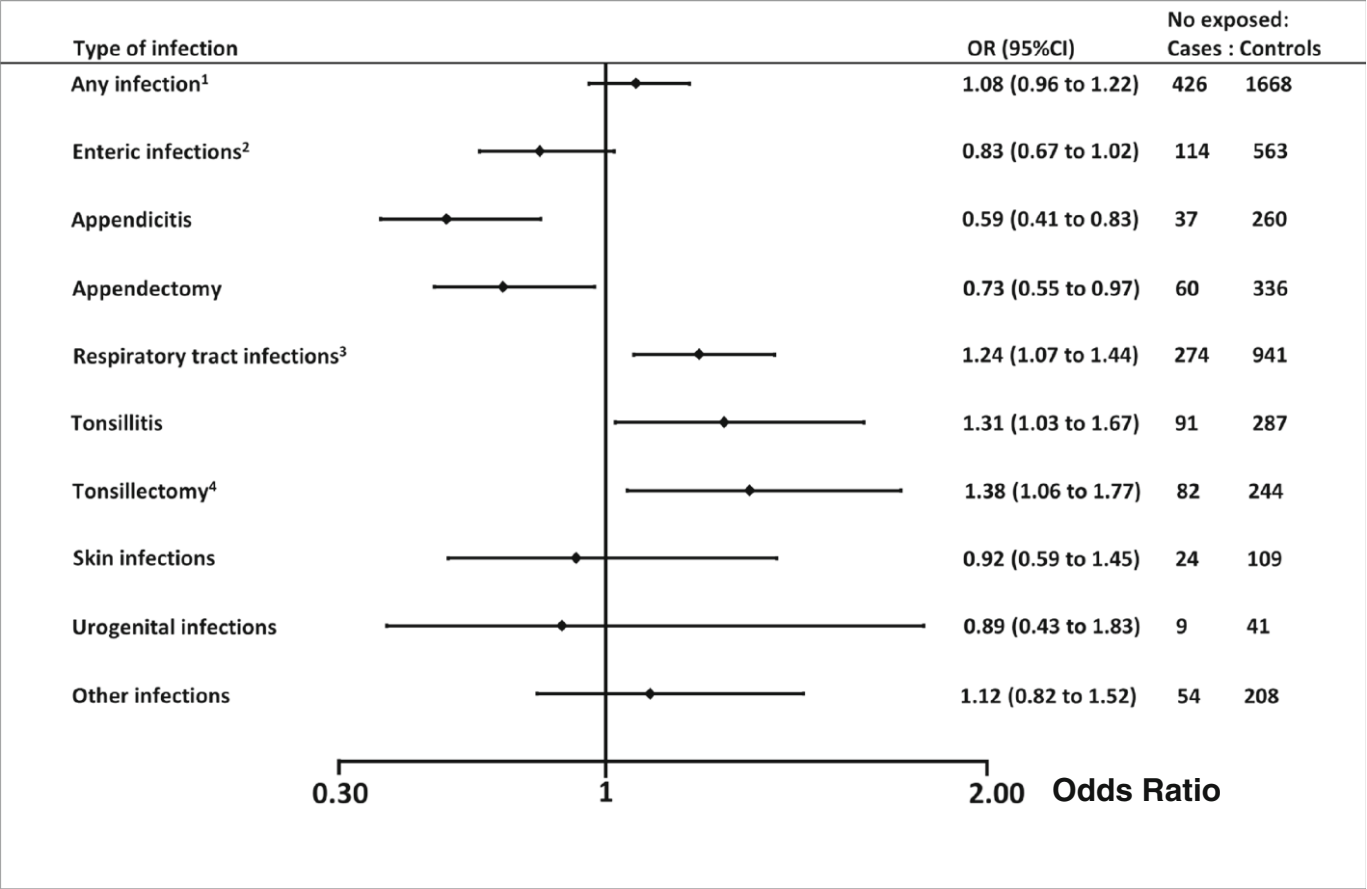
Associations between childhood infections and a later diagnosis of ankylosing spondylitis. Based on a total of 2453 AS cases and 10,257 matched controls where exposure data to infections are based on registered events of an infection in inpatient care before the age of 17 years. ^1^Including all infectious foci, but not the procedures appendectomy or tonsillectomy; ^2^including appendicitis; ^3^including tonsillitis; ^4^also including adenoidectomy. *CI* confidence interval, *OR* odds ratio

Of the AS cases, 426 (17.4 %) had a registered diagnosis of any infection at a time of hospitalisation before the age of 17 years, with the values for the controls being 1668 (16.3 %) (OR 1.08, 95 % CI 0.96–1.22). Figure 1 presents the numbers of exposed cases and controls for each category of infection, and the corresponding ORs.

Appendicitis (cases *n* = 37 (1.5 %) and controls *n* = 260 (2.5 %); OR 0.59, 95 % CI 0.41–0.83) and appendectomy (cases *n* = 60 (2.4 %) and controls *n* = 336 (3.3 %); OR 0.73, 95 % CI 0.55–0.97) were significantly associated with a decreased OR for AS. Respiratory tract infections (cases *n* = 274 (11.2 %) and controls *n* = 941 (9.2 %); OR 1.24, 95 % CI 1.07–1.44) and, in particular, tonsillectomy (cases *n* = 82 (3.3 %) and controls *n* = 244 (2.4 %); OR 1.38, 95 % CI 1.06–1.77) were associated with an increased OR for AS. For enteric infections overall and urogenital infections there were no associations.

For the group registered with both appendicitis and appendectomy (cases *n* = 37 (1.5 %) and controls *n* = 254 (2.5 %)) the OR was 0.60 (95 % CI 0.42–0.85), while for the group registered with only appendectomy but not appendicitis (cases *n* = 23 (0.9 %) and controls *n* = 82 (0.8 %)) the OR was 1.16 (95 % CI 0.73–1.86). For the group registered with both tonsillitis and tonsillectomy (cases *n* = 74 (3.0 %) and controls *n* = 222 (2.2 %)), the OR was 1.38 (95 % CI 1.04–1.78). For the respiratory tract infections, excluding tonsillectomy or tonsillitis (cases *n* = 179 (7.3 %) and controls *n* = 636 (6.2 %)), the OR was 1.20 (95 % CI 1.00–1.43).

The point estimates were of a similar direction and magnitude in all of the sensitivity analyses (Table 2). In the sensitivity analysis only including cases matched 2007–2011 there was no statistically significant difference in the proportions receiving prescriptions of antibiotics in the year prior to matching between cases and controls.

**Table 2:** Sensitivity analyses of the associations between childhood infections and a later diagnosis of ankylosing spondylitis

Including only cases matched at an age of 27 years or older	Cases (*n* = 1757)	Controls (*n* = 7305)	OR	95 % CI
All infections^a^	258 (14.7)	964 (13.2)	1.13	0.97–1.32
Enteric^b^	74 (4.2)	333 (4.6)	0.91	0.70–1.19
Appendicitis	30 (1.7)	169 (2.3)	0.72	0.49–1.07
Appendectomy	46 (2.6)	234 (3.2)	0.82	0.59–1.13
Respiratory tract^c^	159 (9.0)	529 (7.2)	1.27	1.05–1.54
Tonsillitis	49 (2.8)	169 (2.3)	1.16	0.83–1.61
Tonsillectomy^d^	43 (2.4)	151 (2.1)	1.12	0.79–1.59
Skin	15 (0.9)	66 (0.9)	0.93	0.53–1.64
Urogenital tract	6 (0.3)	24 (0.3)	1.02	0.41–2.50
Other	35 (2.0)	113 (1.5)	1.34	0.91–1.97
Including only cases treated with methotrexate, sulphasalazine, and/or TNFi	Cases (*n* = 794)	Controls (*n* = 3288)	OR	95 % CI
All infections^a^	141 (17.8)	553 (16.8)	1.06	0.86–1.31
Enteric^b^	40 (5.0)	185 (5.6)	0.87	0.61–1.25
Appendicitis	12 (1.5)	85 (2.6)	0.58	0.31–1.06
Appendectomy	20 (2.5)	116 (3.5)	0.71	0.44–1.16
Respiratory tract^c^	93 (11.7)	329 (10.0)	1.20	0.93–1.54
Tonsillitis	34 (4.3)	96 (2.9)	1.48	0.98–2.24
Tonsillectomy^d^	27 (3.4)	78 (2.4)	1.42	0.90–2.24
Skin	12 (1.5)	38 (1.2)	1.37	0.71–2.66
Urogenital tract	1 (0.1)	16 (0.5)	0.28	0.037–2.10
Other	14 (1.8)	59 (1.8)	0.991	0.54–1.81
Including only cases matched 2007–2011 and excluding all with immunosuppressive or cytostatic treatment, as well as specific treatment for inflammatory bowel disease, 1 year prior to match	Cases (*n* = 471)	Controls (*n* = 1836)	OR	95 % CI
All infections^a^	96 (20.4)	329 (17.9)	1.16	0.89–1.52
Enteric^b^	26 (5.5)	102 (5.6)	0.92	0.59–1.45
Appendicitis	6 (1.3)	38 (2.1)	0.57	0.24–1.36
Appendectomy	11 (2.3)	48 (2.6)	0.84	0.43–1.63
Respiratory tract^c^	63 (13.4)	191 (10.4)	1.33	0.97–1.81
Tonsillitis	27 (5.7)	67 (3.6)	1.58	0.99–2.53
Tonsillectomy^d^	20 (4.2)	54 (2.9)	1.40	0.82–2.38
Skin	3 (0.6)	21 (1.1)	0.56	0.16–1.90
Urogenital tract	4 (0.8)	8 (0.4)	2.03	0.61–6.78
Other	14 (3.0)	45 (2.5)	1.29	0.70–2.40
Prescription of antibiotics 1 year prior to match	131 (27.8)	478 (26.0)	*p* = 0.446	
Excluding inflammatory bowel disease^e^	Cases (*n* = 2307)	Controls (*n* = 9584)	OR	95 % CI
All infections^a^	405 (17.6)	1555 (16.2)	1.10	0.97–1.25
Enteric^b^	108 (4.7)	528 (5.5)	0.84	0.67–1.04
Appendicitis	35 (1.5)	244 (2.5)	0.59	0.41–0.84
Appendectomy	56 (2.4)	312 (3.3)	0.74	0.55–0.98
Respiratory tract^c^	258 (11.2)	873 (9.1)	1.25	1.08–1.46
Tonsillitis	87 (3.8)	257 (2.7)	1.37	1.07–1.77
Tonsillectomy^d^	78 (3.4)	219 (2.3)	1.42	1.09–1.85
Skin	23 (1.0)	102 (1.1)	0.94	0.59–1.49
Urogenital tract	9 (0.4)	40 (0.4)	0.90	0.44–1.86
Other	53 (2.3)	192 (2.0)	1.18	0.87–1.62

All values are shown as *n* (%)

Exposure data are based on registered events of an infection in inpatient care before the age of 17 years

^a^Including all infectious foci, but not the procedures appendectomy or tonsillectomy; ^b^including appendicitis; ^c^including tonsillitis; ^d^also including adenoidectomy; ^e^excluding all cases/controls with a diagnosis of ulcerative colitis, Crohns’ disease, or any other non-infectious bowel inflammation (see Additional file 1) up until 2 years after the first AS diagnosis of the index case

*CI* confidence interval, *OR* odds ratio, *TNFi* tumour necrosis factor alfa inhibitors

In the analysis stratified by median age at first SpA diagnosis (17–30 years versus 31–46 years) all point estimates were in the same direction and of similar magnitude as in the primary analysis, apart from tonsillitis and tonsillectomy which were stronger in the younger age-groups (age 17–30 years at first SpA diagnosis: tonsillitis, OR 1.15 (95 % CI 1.13–2.94), and tonsillectomy, OR 1.65 (95 % CI 1.21 to 2.26); age 31–46 years at first SpA diagnosis: tonsillitis, OR 0.97 (95 % CI 0.62–1.51), and tonsillectomy, OR 0.94 (95 % CI 0.59–1.51).

The median age for appendectomy was 12 years and for tonsillectomy 9 years (all procedures at an age above 16 years are disregarded). In the analysis stratifying on median age at appendectomy, a stronger association was observed in the age group 0–12 years (OR 0.61, 95 % CI 0.42–0.89) compared to age group 13–16 years (OR 0.95, 95 % CI 0.63–1.43). For tonsillectomy, the association was similar in the stratified age groups (age 0–9 years, OR 1.43 (95 % CI 1.01–2.02), and age 10–16 years, OR 1.35 (95 % CI 0.94–1.93).

## Discussion

### Principal findings

In this study we found that appendicitis in childhood was associated with a lower OR for AS, and that respiratory tract infections were associated with a weak increase in the OR, which was more pronounced for tonsillitis. Our initial hypothesis was that infections in general during childhood, particularly in the enteric and urogenital tracts, would be associated with an increased risk for later development of AS. However, our results did not indicate any such association with either infections in general, or specifically with enteric or urogenital tract infections.

Appendicitis has repeatedly been shown to have a protective association with ulcerative colitis, thought to be linked somehow to the inflammation itself rather than the appendectomy [26, 27]. In this study, the decreased OR observed for appendicitis remained much the same after exclusion of cases and controls with IBD, suggesting that the effect was not conveyed through IBD comorbidity. The point estimates for appendicitis and appendectomy were also conserved throughout the other sensitivity analyses, suggesting a robust effect. For respiratory tract infections, the OR also attained statistical significance; however, the magnitude of the effect was in fact similar to that of enteric infections and urogenital tract infections. The reason that the latter two did not meet statistical significance might be due to the relatively low frequency of these infections, implying that a larger number of cases and controls were needed for respiratory tract infections to reach statistical difference. This may suggest that the strength of the association between respiratory tract infections overall and the risk of developing AS is relatively weak. For tonsillitis and tonsillectomy, the increase in risk for development of AS was consistently stronger than for respiratory tract infections overall in all sensitivity analyses except when only including cases receiving a registered diagnosis of SpA after the age of 26 years.

There are several remarks to make concerning the possible biological interpretation of our findings. First, it is not possible to draw any conclusions concerning causality for the association found between different types of infections and later development of AS. Indeed, since the frequency for all types of infections was relatively low among both the cases and the controls, and the time span between the exposures and the outcome was long, such an effect may be biologically improbable. Still, it cannot be ruled out that the different infections during childhood could induce long-lasting immunological effects that may affect the disease onset in genetically predisposed individuals. If so, then it is also possible that repeated infections in the same individual may enforce such an effect, but the frequencies of readmissions with the same infection were too low to analyse in our study (data not shown). Second, we know that HLA is central to the function of the adaptive immune system, and that different HLA types have been shown to be associated with differences in the response to different pathogens; for example, HLA-B27 has been shown to be related to a higher chance for viral clearance in hepatitis C and a better prognosis in HIV [28, 29]. It is possible that the immune phenotype of AS, including HLA-B27, leading up to the development of AS also conveys a hitherto unknown increase in risk for respiratory tract infections and a decrease in risk for appendicitis. For appendicitis, a genetic component has been suggested but the inflammation is generally believed to be triggered by infections [30]. Third, it is also possible that the similar decrease in risk for both AS and ulcerative colitis, associated with appendicitis, is related to the connection between gut inflammation and AS. A fourth possibility is that appendicitis and respiratory tract infections, or the treatment of such, alters the gut flora, leading to a pathological imbalance between microbes and immune phenotype, which in turn affects the risk of developing AS [16]. In our stratified analysis, the association with appendicitis was stronger in the age group 0–12 years, which may suggest that the possible biological or immunological impact of the inflammation is of greater importance if occurring early in life.

### Limitations

First, there may be an issue of reverse causality since the time point of disease onset for AS cannot be precisely determined from the healthcare registers used. We performed one sensitivity analysis in order to further separate the time period for exposures to infections from the time point of disease onset which resulted in similar point estimates, supporting the temporal order of the infections occurring prior to the AS onset. However, this temporal separation of exposures and disease onset also precludes the possibility of detecting any association between infections and AS onset that are close in time, as in the case of reactive arthritis. Second, we only have reliable data for infections diagnosed in inpatient care, reflecting more severe infections. This will underestimate the frequency of infections (e.g. enteric, respiratory, or urogenital) which are normally treated in outpatient care or not treated at all, and it cannot be assumed that the “tip of the iceberg” spectrum of infections that we detect are representative of all childhood infections. By contrast, appendicitis is routinely treated in inpatient care; hence, this result is unlikely to be biased by any selection with regard to exposure.

### Strengths

First, this is the first study directly investigating the relationship between infections during childhood and later development of AS. Second, the high coverage of the national registers minimises selection bias, and our previous validation study of the AS diagnoses in the register support the validity and generalisability of the results [21, 25].

## Conclusions and implications

Appendicitis during childhood was associated with a decreased risk for adult AS, whereas respiratory tract infections were associated with an increased risk. Further studies are required to determine if these associations are a result of the immune phenotype leading to AS, or whether the infections in fact alter the risk for AS. Such studies would preferably take into account HLA-B27 status, but also other risk genes and wider range of infectious exposures, not limiting the latter to infections at hospitalisation.

## Additional file

Additional file 1: Table S1. ICD-10 codes, procedure codes, and ATC codes used in the study to define rheumatic diseases, comorbidities, infectious exposures, procedures, and medications. (DOCX 21 kb)

## Abbreviations

AS: Ankylosing spondylitis
ATC: Anatomical therapeutic chemical
CI: Confidence interval
HLA-B27: Human leukocyte antigen 27
IBD: Inflammatory bowel disease
ICD: International Classification of Diseases
OR: Odds ratio
SpA: Spondyloarthritis
SRQ: Swedish Rheumatology Quality Register
TNFi: Tumour necrosis factor alpha inhibitors
