# Supplementary material for: Childhood hospitalisation with infections and later development of ankylosing spondylitis: a national case-control study
Source: Arthritis Res Ther. 2016 Oct 22;18:240. doi: 10.1186/s13075-016-1141-8 (PMC5075148; doi:10.1186/s13075-016-1141-8)
Supplement: Additional file 1: Table S1. — ICD-10 codes, procedure codes, and ATC codes used in the study to define rheumatic diseases, comorbidities, infectious exposures, procedures, and medications. (DOCX 21 kb) [file 13075_2016_1141_MOESM1_ESM.docx]

Additional file 1: Table S1. ICD-10 codes, procedure codes and ATC-codes used in the study to define rheumatic diseases, comorbidities, infectious exposures, procedures and medications.

| **Diagnoses** | **ICD 10** | **ICD 9: 1987-1996** | **ICD 8: 1969-1986** |
| --- | --- | --- | --- |
| **Rheumatic diseases** | | | |
| Ankylosing spondylitis | M45 | 720A | 712,40 |
| Juvenile arthritis | M08, M09 | 714D | 712,0 |
| Rheumatoid arthritis | M05, M060, M062, M063, M068, M069, M123 | 714A, 714B, 714C, 714W, 719D | 712.10; 712.20; 712.38; 712.39 |
| Polyarthritis | M130 | 714X, 716F | 715.99 |
| Psoriatic arthritis | L405, M070, M071, M072, M073 | 696A, 713D | 696.00 |
| Systemic Lupus Erythematosus | M320, M321, M328, M329 | 710A | 734.10 |
| Undifferentiated spondyloarthritis | M460, M461, M468, M469 | 720B, 720C, 720W, 720X | 713.13; 726.99 |
| Reactive arthritis | M013-M029, M03 | 711B, 711D-711W |  |
| Spondyloarthritis associated with IBD | M074-M075 |  |  |
| **Other inflammatory diseases** | | | |
| Inflammatory bowel disease | K50-K51 | 555-556 | 563.00, 563.10, 569.02 |
| Other non-infectious bowel inflammation | K52 | 558 | 563.98, 563.99 |
| Psoriasis | L40 | 696 | 696 |
| Iridocyclitis | H20, H221 | 364A-364B |  |
| **Infections** | | | |
| All infections | A00-B99, G00-G02, G04.2, G05-G07, H66-H67, H70, J00-J22, J32, J34.0, J35, J36, J38.3, J39.0-J39.1, K10.2, L00-L08, M00-M01, M46.2-M46.5, M86, N10, N30.0, K35-K38 | 001-139, 320-322, 382-383, 460-466, 475, 480-487, 526E-526F, 590, 680-686, 711A, 711E, 730, 790H, 540-543 | 000-136, 320, 322, 381-383, 460-466, 470-474, 480-486, 500.99, 501, 526.4, 590, 680-686, 710, 720, 782.9, 540-543 |
| Enteric infections | A00-A09, K35-K38 | 001-009, 540-543 | 000-009, 540-543 |
| Respiratory infections | A37, A38, B26, H66-H67, H70, J00-06, J32, J34.0, J35, J36, J39.0-J39.1, J09-J22 | 033, 034, 072, 460-465, 475, 382-383, 463, 466, 480-487 | 033, 034, 072, 381-383, 460-465, 501  466, 470-474, 480-486, 500.99 |
| Skin infections | A46, B01, B05, B06, L00-L08 | 035, 052, 055, 056, 680-686 | 035, 052, 055, 056, 680-686 |
| Urogenital infections | A50-A64, N10, N30.0 | 090-099, 590, 595A | 090-099, 590, 595.00 |
| Appendicitis | K35-K38 | 540-543 | 540-543 |
| Tonsillitis | J03, J35 | 034, 463 | 034, 500.99 |
| **Procedures** | **Code 1997-** | **Code 1963-1996** | |
| Appendectomy | JEA00, JEA01, JEA10 | 4510, 4511, 0058 | |
| Tonsillectomy | EMB10, EMB15, EMB20, EMB30 | 2710, 2720, 2730 | |
| **Drug Group** | **ATC-code** | **Drug name** | |
| Disease modifying anti-rheumatic drugs | A07EC01 | Sulphasalazine | |
|  | L01BA01, L04AX03 | Methotrexate | |
| TNF-α-inhibitors | L04AB01 | Etanercept | |
|  | L04AB02 | Infliximab | |
|  | L04AB04 | Adalimumab | |
|  | L04AB05 | Certolizumabpegol | |
|  | L04AB06 | Golimumab | |
| Immunosuppressive drugs | L04AA | Selective immunosuppressive drugs | |
|  | L04AC | Interleukine inhibitors | |
|  | L04AD | Calcineurine inhibitors | |
|  | L04AX | Other immunosuppressive drugs | |
| Cytostatic/cytotoxic drugs | L01AA01 | Cyclophosphamide | |
|  | L01AA02 | Chlorambucile | |
|  | L01BB02 | Mercaptopurine | |
|  | L01XC02 | Monoclonal antibody based cytostatic drugs | |
| Drugs against enteric inflammation | A07EA | Glucocorticoids for local enteric use | |
|  | A07EC | Aminosalicylic derivatives | |
| Antibiotics | A07A | Anti-infective drugs for enteric infections | |
|  | G01 | Anti-infective drugs for gynaecological infections | |
|  | J01-J05 | Antibacterial, antimycotic and antiviral drugs | |
|  | P01-P02 | Drugs against protozoic and worm infections | |
|  | S01A, S02A, S02C, S03CA04 | Topical drugs against eye and ear infections | |
